# Transcallosal connectivity of the human cortical motor network

**DOI:** 10.1007/s00429-016-1274-1

**Published:** 2016-07-28

**Authors:** Kathy L. Ruddy, Alexander Leemans, Richard G. Carson

**Affiliations:** 1 School of Psychology, Queen’s University Belfast, Belfast, BT7 1NN UK; 2 Trinity College Institute of Neuroscience and School of Psychology, Trinity College Dublin, Dublin, Ireland; 3 Neural Control of Movement Lab, Department of Health Sciences and Technology, ETH Zurich, Y36 M 12, Winterthurerstrasse 190, 8057 Zurich, Switzerland; 4 Image Sciences Institute, University Medical Center Utrecht, 85500 Utrecht, The Netherlands

**Keywords:** Transcallosal, Structural connectivity, Motor cortex, Constrained Spherical deconvolution, White matter, Primary motor cortex, Supplementary motor area, Dorsal premotor cortex, Corpus callosum

## Abstract

The organisational and architectural configuration of white matter pathways connecting brain regions has ramifications for all facets of the human condition, including manifestations of incipient neurodegeneration. Although diffusion tensor imaging (DTI) has been used extensively to visualise white matter connectivity, due to the widespread presence of crossing fibres, the lateral projections of the corpus callosum are not normally detected using this methodology. Detailed knowledge of the transcallosal connectivity of the human cortical motor network has, therefore, remained elusive. We employed constrained spherical deconvolution (CSD) tractography—an approach that is much less susceptible to the influence of crossing fibres, in order to derive complete in vivo characterizations of white matter pathways connecting specific motor cortical regions to their counterparts and other loci in the opposite hemisphere. The revealed patterns of connectivity closely resemble those derived from anatomical tracing in primates. It was established that dorsal premotor cortex (PMd) and supplementary motor area (SMA) have extensive interhemispheric connectivity—exhibiting both dense homologous projections, and widespread structural relations with every other region in the contralateral motor network. Through this in vivo portrayal, the importance of non-primary motor regions for interhemispheric communication is emphasised. Additionally, distinct connectivity profiles were detected for the anterior and posterior subdivisions of primary motor cortex. The present findings provide a comprehensive representation of transcallosal white matter projections in humans, and have the potential to inform the development of models and hypotheses relating structural and functional brain connectivity.

**Electronic supplementary material:**

The online version of this article (doi:10.1007/s00429-016-1274-1) contains supplementary material, which is available to authorized users.

## Introduction

The corpus callosum (CC) is by far the largest white matter fibre bundle in the human brain. Forming a bridge between the two hemispheres, it serves as a fundamental channel for interhemispheric communication, including that which mediates bimanual coordination (Brinkman 1984) and the learning of new motor skills. Degeneration of the corpus callosum is also a consistent feature of amyotrophic lateral sclerosis (ALS) (Filippini et al. 2010), and the microstructural pathology of transcallosal projections within the corticomotor network provides a candidate imaging biomarker (Chapman et al. 2014). While specific functional roles of the CC were first ascribed on the basis of motor deficits observed in patients with callosal lesions, and in those who had undergone partial callosotomy (Caillé et al. 2005; Eliassen et al. 1999, 2000; Jeeves et al. 1988; Preilowski 1972), neuroimaging is now being used to infer causal relationships between transcallosal structural connectivity and interhemispheric functional connectivity (O’Reilly et al. 2013). Several cortical brain regions are known to contribute to different aspects of motor planning, learning and execution, and with many functional sub-divisions emerging, there is an increasing need to understand the network interactions between motor regions in one hemisphere with both their homologous counterparts, and heterogenous regions in the opposite hemisphere. As there is increasing evidence that brain structure predicts function (e.g., Honey et al. 2010), the facility to extract reliable estimates of white matter organisation is of profound importance in resolving anatomical constraints upon functional motor network connectivity.

**Fig. 1:**
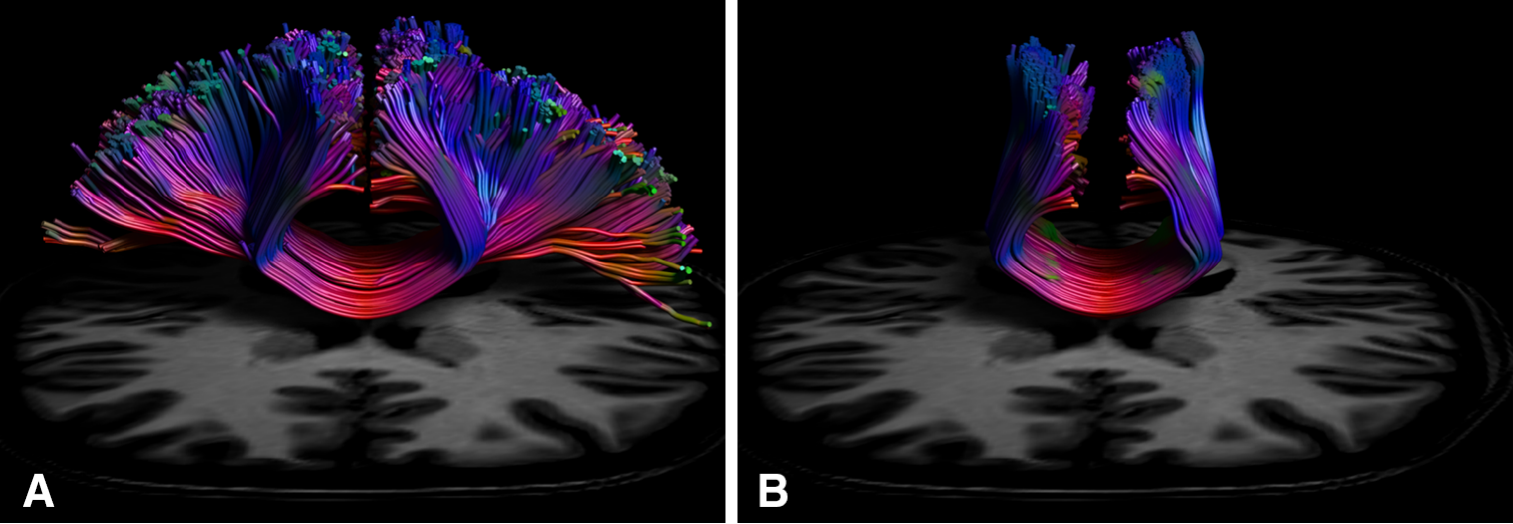
Transcallosal dorsal premotor cortex (PMd) tracts reconstructed using CSD and DTI. Both panels show PMd–PMd reconstructed streamlines for the same ROI in the same participant. The tractography algorithm for panel **A** was based upon constrained spherical deconvolution (CSD). For panel **B** the traditional diffusion tensor model was used

Although anatomical tracer studies in non-human primates have contributed greatly to our understanding of transcallosal structural connectivity, given the invasive nature of these procedures, there are no equivalent data for humans. Furthermore, the general assumption that patterns of connectivity thus derived extend directly to human brain networks cannot necessarily be sustained (Mantini et al. 2013). At first glance diffusion weighted (DW) neuroimaging—which permits the in vivo quantification of white matter in human brains, appears to circumvent this issue. The diffusion tensor model that is typically applied to DW imaging data in this context is, however, inadequate for regions that contain complex architectures such as crossing fibres (Alexander et al. 2001; Jeurissen et al. 2013; Tuch et al. 2002). The lateral cortical projections of the corpus callosum connecting motor regions are particularly susceptible to this limitation, due to the presence of crossing fibres from other bundles such as the superior longitudinal fasciculus (SLF) and the corticospinal tracts (Jeurissen et al. 2011). As many of the transcallosal projections between nodes of the cortical network are therefore beyond the reach of the diffusion tensor model, previous estimates of structural connectivity have been limited to the subset of fibre trajectories that could be detected using this method. Although recently developed high-angular resolution DW technologies have been used to generate global indices of callosal connectivity (Chao et al. 2009; Jarbo et al. 2012), the transcallosal connections of the cortical *motor* network have yet to be fully revealed. In the current investigation we used constrained spherical deconvolution (CSD) tractography (Tournier et al. 2007; Jeurissen et al. 2011) to characterise transcallosal fibre trajectories connecting homologous and non-homologous regions of the cortical motor network. CSD provides indices of the distribution of fibre orientations within a given voxel, which improves tract reconstructions in regions with crossing fibres (see Fig. 1). In a recent investigation combining post-mortem human brain dissections with CSD based tractography, Vergani (Vergani et al. 2014) reported that the two methods demonstrated good coherence, with all fibre bundles identified by dissection also delineated by CSD based tractography. We provide detailed characterizations of both homologous and non-homologous transcallosal fibre trajectories in vivo, and profile the anatomical connectivity revealed for key nodes of the cortical motor network. The intent is to provide a comprehensive resource for future investigators to consult when developing hypotheses regarding functional connectivity, such that realistic neuroanatomical constraints may be applied. Consideration is also given to the potential functional implications that arise from the profiles of anatomical connectivity, thus, revealed.

## Materials and methods

### Participants

Forty-three neurologically healthy volunteers (aged 22.5 ± 2.9 SD, 28 female), who were all right handed according to the Edinburgh handedness inventory (Oldfield 1971), gave informed consent to procedures that were conducted in accordance with the Declaration of Helsinki, and approved by the relevant Queen’s University Belfast and Trinity College Dublin Ethics Committees. The sample was limited to right handed volunteers, as white matter asymmetries related to handedness have previously been reported in the precentral gyrus (Buchel et al. 2004).

### Image acquisition and processing

Diffusion weighted images were acquired on a 3T Philips Achieva magnetic resonance scanner, using an eight channel head coil. The sequence consisted of single shot echo planar imaging (EPI) with a slice thickness of 2.29 mm, repetition time = 9994 ms, echo time = 73 ms, number of diffusion directions = 61, *b* value = 1500 s/mm^2^, number of slices = 60 (transverse), in-plane resolution 2.3 × 2.3 mm^2^, with a field of view of 258 mm (RL) × 258 mm (AP) × 138 mm (FH). Scan duration was approximately 14 min.

Data were processed using ExploreDTI (Leemans et al. 2009). Images were corrected for head movement and eddy currents using the procedure described in Leemans and Jones (2009). Tensor estimation was performed using the iteratively reweighted linear least squares approach (Veraart et al. 2013). Fibre trajectories were computed with CSD based tractography (Tournier et al. 2007) using recursive calibration of the response function to optimise the estimation of the fibre orientation distribution (FOD) functions (Tax et al. 2014). A uniform grid of tractography seed points at a resolution of 2 × 2 × 2 mm^3^ was used with an angle threshold of 30 degrees, an FOD threshold of 0.1, and maximum harmonic order of eight. The median number of streamlines computed for each participant was 55,221 (IQR 8665).

### Connectivity analysis

**Fig. 2:**
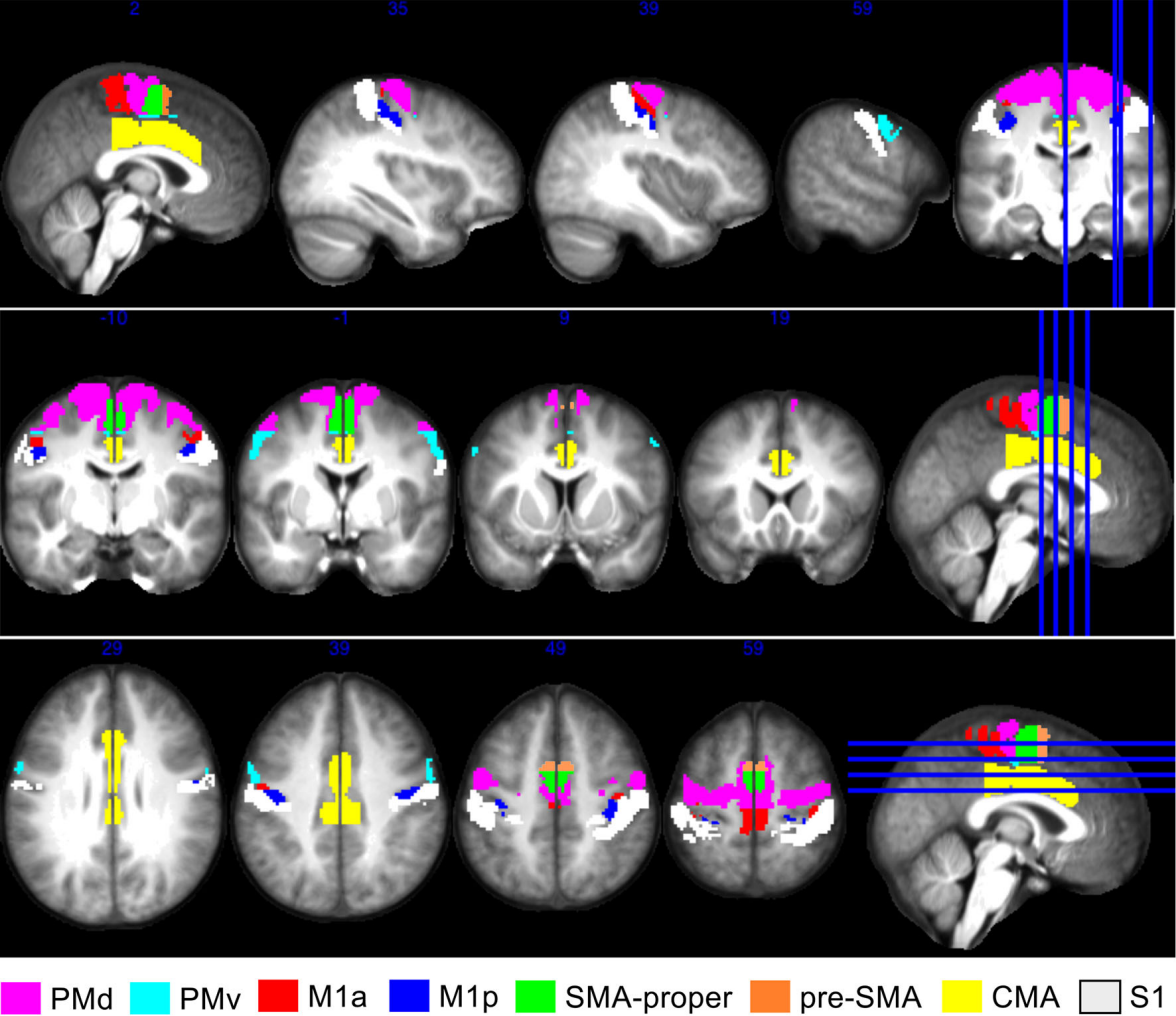
Regions of interest used for network connectivity analysis. ROIs are displayed on an averaged T1 from a subset of participants

Cortical motor regions of interest (ROIs) selected for connectivity analysis included posterior and anterior primary motor cortex (M1a and M1p), dorsal and ventral premotor cortex (PMd and PMv), supplementary motor area proper (SMA proper) and pre-supplementary motor area (pre-SMA), primary sensory cortex (S1), and the cingulate motor area (CMA). Regions of interest for M1a, M1p, premotor cortex and primary sensory cortex were derived from the Juelich histological atlas (Eickhoff et al. 2005, 2006, 2007). We then subdivided this ‘premotor’ region along *z* = 48, consistent with reports of the anatomical boundary separating PMd and PMv (Tomassini et al. 2007). The supplementary motor area (SMA) region was derived from the Harvard-Oxford Cortical atlas, and subdivided along *y* = 0, in order to demarcate pre-SMA and SMA proper (Zhang et al. 2011). The cingulate motor area in the mid cingulate cortex was demarcated as the portion of the Harvard Oxford (anterior and posterior) cingulate cortex regions that lay within the furthermost anterior and posterior coordinates for the cingulate motor region reported by Amiez and Petrides (2012). A custom atlas comprising these eight motor cortical regions (in both hemispheres) was composed, with care being taken to ensure that there was no overlap between regions derived from the Juelich and Harvard Oxford Cortical atlases (Fig. 2). This was then warped to the ICBM Mori FA template using the ELASTIX approach (Klein et al. 2010) with non-rigid (B-splines) registration, and applied to the data obtained from each participant. We have made this custom atlas freely available as part of the *ExploreDTI* toolbox (Leemans et al. 2009).

Connectivity analysis was carried out by determining bilateral pairs of regions, and extracting only the fibre pathways that passed through both. This was performed for homologous and non-homologous motor regions for all 43 participants. Due to the very large number of separate tract bundles that were extracted, manual removal of spurious streamlines (false positives) was not feasible. Necessarily, when a standardised template is applied to each dataset, variability across participants will also give rise to the possibility of false negatives. This is, however, an inherent limitation of tractography. Reconstructed fibre trajectories for each individual were quantified in terms of the (median) fractional anisotropy (FA), mean diffusivity (MD), and radial diffusivity (RD), which are all measures that reflect the directional coherence of intracellular water diffusion. These were computed by averaging all the points along the fibre trajectories of a white matter bundle connecting specific ROIs. Additionally, CSD affords quantification of the apparent fibre density (AFD) for any given fibre bundle; a highly sensitive measure of microstructural organisation that is effective even in regions with complex fibre architecture (Dell’Acqua et al. 2012; Raffelt et al. 2012). As raw metrics of the number of streamlines should be approached with caution (Jones et al. 2013), for instances in which number of streamlines data are presented, the absolute values have been normalised to account for volume differences in the ROIs, whereby the number of reconstructed streamlines connecting two regions was divided by the total number of streamlines passing through each of the two ROIs in the pair being considered, and expressed as a percentage of this total. Thus, this takes account of the possibility that larger regions will simply have a greater number of reconstructed streamlines.

### Data analysis

As the FA values in our dataset deviated from normality both across the entire brain and within local tracts, a transformation to the F distribution was first performed following the method described by Clement-Spychala et al. (2010) and Cascio et al. (2013) in order to increase the symmetry of the sample distribution. As an additional means of achieving robustness of inferential tests, equivalent analyses were also performed using rank transformations of the FA values. As there is not yet a theoretically derived transform for AFD values, the inferential analyses for this measure were based upon rank transformed values.

Mixed effects models (Pinheiro and Bates 2006) employing restricted maximum likelihood (REML) estimation and an unstructured covariance matrix (‘participant’ designated as a random effect) were conducted to examine variations in each dependent measure (AFD and FA) across fibre bundle pathways. A limited set of planned contrasts were assessed using the least squares means approach. The false discovery rate (FDR) correction for multiple comparisons was used to adjust the corresponding alpha levels. Inferential statistics for comparison of fibre trajectories connecting homologous pairs of regions with those of other homologous pairs were conducted separately from those for non-homologous pairs of regions. As the outcomes of analyses based on the F-transform of the FA values and the rank transformed FA values were equivalent in almost every instance (26/28 homologous contrasts, 204/210 non-homologous), and to facilitate comparison with AFD, the presentation given in the Results section below is derived from the results of statistical comparisons based on rank transformed data. In the interests of brevity, we herein report medians and confidence intervals (95 %) derived using a bootstrapping approach (supplementary Table 1). A detailed presentation of the results of the corresponding inferential analyses is available on-line.

As the total number of reconstructed fibre tracts passing through an ROI inevitably included many pathways that were not connected to any of the eight ‘motor’ ROIs in the opposite hemisphere, we aimed to quantify separately the connectivity of each region with regards to ‘interhemispheric motor’ pathways, and ‘other’ pathways. The ‘other’ trajectories most likely correspond to ipsilateral intrahemispheric motor connections or connections with other ‘non-motor’ regions of the brain. With the aim of further characterising this demarcation, an additional network connectivity analysis was undertaken whereby all eight ROIs in each hemisphere were first merged into a single large ROI. The connections from this composite ROI to each of the eight ROIs in the opposite hemisphere were then enumerated (separately) to provide an estimate of the total number of reconstructed tract pathways. This step allowed the number of reconstructed pathways passing through a given region that were connected to ‘motor’ regions in the opposite hemisphere, to be expressed as a proportion of the total number of reconstructed tract pathways. Additionally, this analysis allowed quantification of the number of trajectories that passed through more than one ROI in the same hemisphere prior to crossing the corpus callosum. The extent of this duplicity was quantified by subtracting the number of connections between the composite ROI of one hemisphere and a target region in the other hemisphere, from the total number of connections between the target region and the eight ROIs in the opposite hemisphere—when these were considered separately. This was performed bidirectionally, from right to left hemisphere ROIs and then repeated in the opposite direction. The resulting values were then averaged across right and left, and are reported in Fig. 4.

## Results

### Cortical territories displaying connectivity with the contralateral hemisphere

**Fig. 3:**
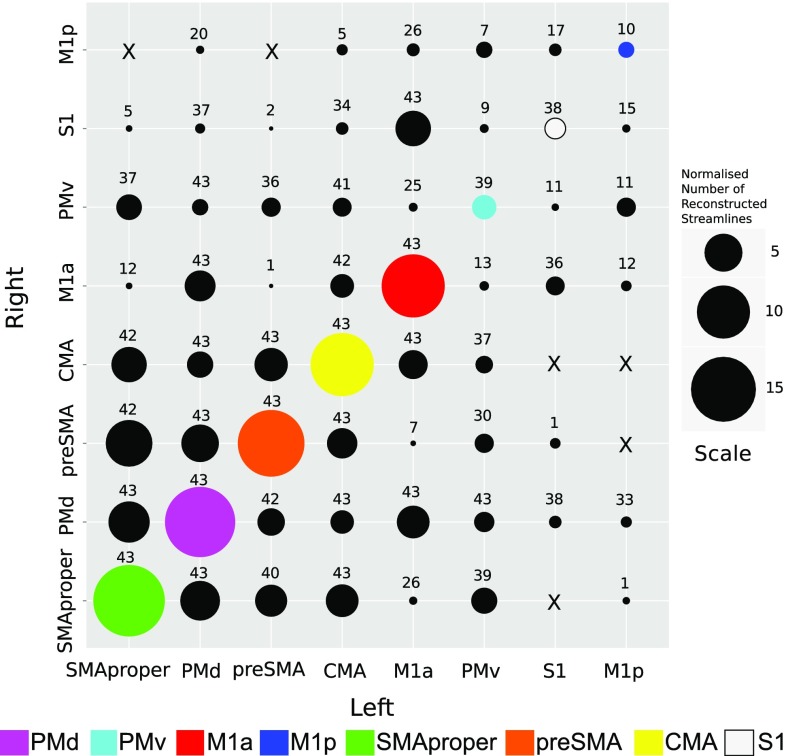
Balloon plot illustrating the normalised quantity of reconstructed streamlines. Data are presented for both left–right and right–left pairs. The numbers presented are normalised to account for volume differences between ROIs (see "Materials and methods"). The number of participants (from a total of 43) for whom tracts were delineated is shown above each circle. The colour codes for homologous pairs—*lower left* to *upper right* diagonal, identify each ROI (and are used in all subsequent figures). All corresponding values for the normalised number of reconstructed streamlines are given in supplementary Table 1

**Fig. 4:**
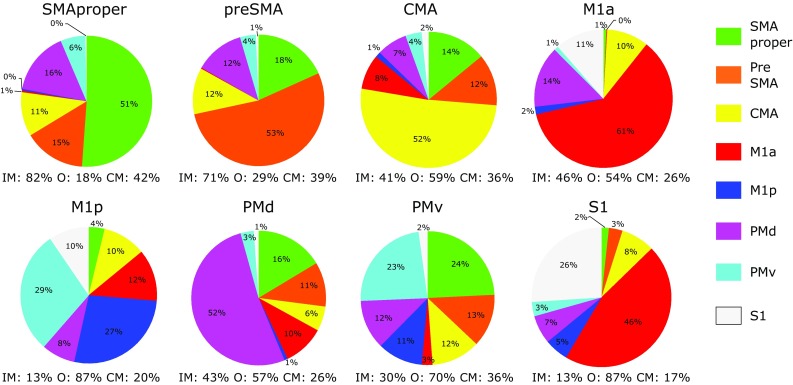
Reconstructed streamlines passing through motor regions in the contralateral hemisphere. Each *pie* represents the composition of the named motor region in terms of reconstructed streamlines passing through the ROI that also exhibit transcallosal connectivity with motor regions in the opposite hemisphere (mean of right/left and left/right). Consequently, the pie represents only the interhemispheric motor streamlines (indicated by IM), which constitute a subset of the total number of reconstructed streamlines that were detected passing through the region. The remainder, which may pass through non-motor regions or correspond to intrahemispheric projections, are quantified and indicated below each pie by O (other). As many streamlines pass through two or more neighbouring regions on their trajectory prior to crossing the corpus callosum, some may be ‘counted’ more than once. The percentage of streamlines for a given region that may have been counted multiple times is denoted ‘CM’. All quantities of reconstructed streamlines have been normalised to account for volume differences (see "Materials and methods")

The number of participants for whom fibre tracts connecting homologous and non-homologous regions were detected, and the associated normalised quantity of reconstructed pathways, are shown in Fig. 3 and supplementary Table 1. To further characterise the transcallosal structural connectivity represented by these fibre pathways, we delineated separately for each node the composition of the region, in terms of the percentage of all trajectories passing through the ROI that were connected to each of the other motor regions in the opposite hemisphere (Fig. 4).

### Connectivity between homologous motor regions

**Fig. 5:**
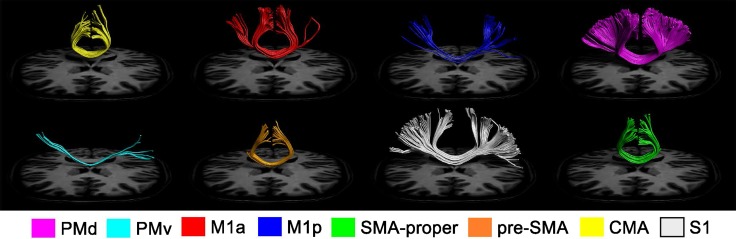
Reconstructed streamlines connecting homologous motor regions. Transcallosal tracts passing through homologous regions on both left and right hemispheres are displayed for one representative participant. For the purpose of graphical display only, spurious tracts (false positives) were removed using ‘not’ gates based on prior anatomical knowledge

Streamlines connecting homologous PMd, M1a, CMA, SMA proper, and pre-SMA regions were present in all 43 participants. With respect to PMv, homologous transcallosal tracts were evident in 39/43 participants. 38 of the 43 participants exhibited fibre tracts connecting homologous S1. In only 10 of 43 individuals were tracts connecting homologous M1p detected. The quantity of reconstructed streamlines (Supplementary Table 1) was greatest between homologous SMA proper regions (Med 18.85, CI 16.06–20.05), followed closely by homologous PMd regions (Med 18.19, CI 15.94–20.68). The smallest number of transcallosal streamlines was detected between homologous M1p–M1p (Med 0.96, CI 0.59–2.05). This is in contrast with the much denser homologous connectivity of M1a (Med 14.69, CI 13.65–15.56). 95 % confidence intervals are provided in supplementary Table 1 to allow comparisons to be made between different pairs of ROIs. Graphical reconstructions of the computed homologous motor fibre trajectories for one representative participant are displayed in Fig. 5.

### Microstructural organisation

**Fig. 6:**
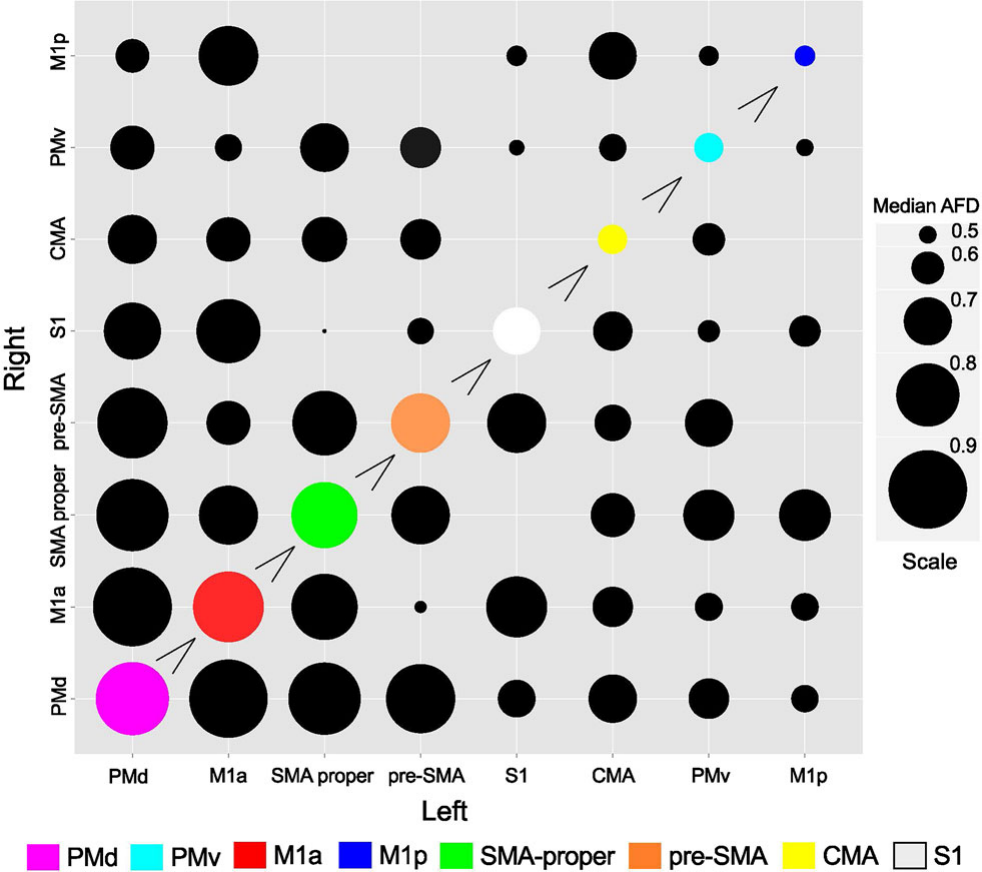
Balloon plot illustrating AFD values obtained for interhemispheric fibre bundles. The “*greater than*” symbol indicates that the homologous connections represented by the larger (*coloured*) *circle* exhibit median AFD values that are greater than those represented by all successive smaller (coloured) *circles*

With respect to homologous tracts, the median apparent fibre density (AFD) values obtained for PMd were significantly larger than those for all other regions with the exception of M1a (all *p* < 0.001). The values for M1a were significantly greater than those for tracts connecting homologous SMA proper, preSMA, S1, CMA, PMv and M1p (all *p* < 0.017). The pathways projecting between left and right M1p were characterised by lower AFD values than those of all other homologous connections with the exception of CMA (all *p* < 0.011) (Fig. 6). For results of inferential statistics see supplementary Table 2.

The median FA values observed for homologous PMd fibre tracts were significantly larger than those obtained for every other homologous pair (all *p* < 0.021). In addition, the FA values that characterised SMA proper to SMA proper projections were greater than those of homologous S1, CMA, PMv and M1p (all *p* < 0.021). Homologous pre-SMA fibre tracts demonstrated FA values that did not differ significantly from those of SMA proper or M1a; however, they were significantly greater than those of homologous S1, CMA, PMv and M1p (all *p* < 0.001). The FA values for tracts connecting M1p were lower than those of every other homologous pair except CMA (all *p* < 0.011). Median values and associated confidence intervals for fibre density (AFD) and fractional anisotropy (FA), obtained for homologous and non-homologous pairs of streamline bundles, are presented in supplementary Table 1. The details of all corresponding inferential analyses are presented in supplementary Table 3.

## Discussion

Given the data presented herein, it is clear that pronounced differences exist in the degree of structural interhemispheric connectivity that characterises non-primary areas of the cortical motor network and regions that are more directly related to movement execution and sensory function. Although the reconstructed fibre trajectories passing through a designated ROI, as derived by tractography, do not correspond to individual neurons as traced by the injection of compounds which highlight the specific locations at which their axons terminate, it is nonetheless striking that the patterns of connectivity identified in the current investigation resemble closely those derived from neuroanatomical studies conducted in non-human primates (Boussaoud et al. 2005; Fang et al. 2008; Marconi et al. 2003; Rouiller et al. 1994). For instance, it has previously been reported that in non-human primates the largest proportions of interhemispheric fibres connecting to M1, SMA, PMd and PMv originate in their homologous region in the contralateral hemisphere (Dancause et al. 2007; Rouiller et al. 1994). Our findings in humans are in agreement with this literature with respect to the normalised number of streamlines connecting homologous PMd, SMA proper, pre-SMA, CMA, M1a, and M1p. Additionally, we observed that the density of PMd–PMd transcallosal connectivity as assessed by CSD based tractography, was markedly greater than that which characterised any other pair of nodes within the defined cortical network—a finding that is consistent with axon labelling studies performed in monkeys (Boussaoud et al. 2005; Fang et al. 2008; Marconi et al. 2003).

The extended interhemispheric motor connectivity of PMd exceeds that of any other node. In most instances the connections to PMd from non-homologous regions exhibited the most coherent and dense microstructural organisation, as reflected by AFD (and FA) values. Indeed, there are also theoretical grounds on which to conclude that the extensive connectivity of PMd reflects demands imposed by the underlying physiology. On the basis of studies employing resting state fMRI it is known that homotopic voxels in the left and right hemispheres exhibit correlated spontaneous fluctuations in the blood oxygenation level dependent (BOLD) response. Interestingly, this tendency for homotopic regions to exhibit strong functional connectivity is expressed least among the most ‘higher order’ regions of the adult human brain. It is most prevalent for visual, motor, somatosensory and subcortical regions. Homotopic regions in the prefrontal cortex, particularly dorsolateral and ventrolateral regions involved in language, attention and cognition, exhibit lower coherence (Zuo et al. 2010). This may in part reflect lateralization of these functions. As the prefrontal cortex is not directly connected with descending motor efferents, all prefrontal influences upon motor control are mediated via the premotor cortex (Badre and D’esposito 2009). By virtue of its rich interhemispheric connectivity, PMd, thus, gives bilateral effect to the lateralised functions of the prefrontal cortex. The high degree of non-homologous structural connectivity between PMd and all other nodes of the cortical motor network revealed by the present study is consistent with this role.

Rather than investigating the primary motor cortical region (M1) as a single entity, we chose to consider the anterior and posterior subdivisions separately. Geyer et al. (1996) has described cytoarchitectural, neurochemical and functional differences between the two regions. Additionally, studies in non-human primates have illustrated differences in ipsilateral anatomical connectivity between anterior and posterior divisions of M1, whereby the posterior subdivision is connected primarily to somatosensory regions and the anterior subdivision to premotor cortex (Stepniewska et al. 1993). Corresponding evidence concerning the functional properties of the two regions in humans is scarce. It has been proposed that there are two spatially distinct fields in M1—one related to the preparation of movement, the other to the generation of motor output (Kawashima et al. 1994). An emerging view from the non-human primate literature is that posterior M1 has greater involvement in motor execution, particularly in complex tasks that require a high level of manual dexterity (Rathelot and Strick 2009). The unique functions of M1a remain to be determined. In the current data, PMd, SMA proper and preSMA, regions known to be involved in early phases of motor preparation and planning, exhibited much greater callosal fibre density than posterior primary motor cortex (M1p). As it is known that tractography algorithms exhibit preferential pathway termination at the crown of gyri rather than the fundi of sulci, this finding should be approached with caution—as most of M1p is deeply buried in the central sulcus. Although it has been argued that regional variations in the differentiation of fibres by tractography reflect such neuroanatomical phenomena (Chen et al. 2013; Nie et al. 2012), invasive tracing experiments in primates show (in general) a complete lack of bias towards gyral or sulcal regions (Markov et al. 2014). It is therefore interesting that our finding of low M1p transcallosal connectivity mirrors the results of primate tracer investigations, in which low density (but existent) callosal connections between regions on the bank of the central sulcus that correspond to M1p have been described (Rouiller et al. 1994). The scope for direct interhemispheric interactions via callosal pathways is believed to decrease progressively along a functional gradient that culminates in those regions that have the most prominent role in generating motor output (Carson 2005). In the context of bimanual movement, it has been proposed previously that this organisation is consistent with the requirement that inter-hemispheric interference at the level of execution is minimised, while mutual “cross-talk” in relation to movement planning is promoted (Liu et al. 2002). It is notable, therefore, that pathways connecting homologous anterior primary motor cortex (M1a) exhibited fibre densities that were comparable to those observed for homologous PMd, SMA proper and preSMA connections. Indeed, there were pronounced differences between M1a and M1p in relation to fibre density, fractional anisotropy and patterns of interhemispheric connectivity. The point is, thus, reinforced that M1 is not a homogenous single region. In light of our finding that patterns of connectivity for M1a resemble PMd, SMA proper and preSMA, a role more closely linked to motor planning than to movement execution is suggested. Necessarily, however, in investigations of functional and structural connectivity, this subdivision should be given explicit consideration.

Of all non-homologous transcallosal reconstructed fibre pathways passing through SMAproper, preSMA, PMd and CMA, only a very small proportion demonstrated connectivity with contralateral M1a or M1p. This is in agreement with previous reports from primate studies (Fang et al. 2008; Jürgens 1984; Rouiller et al. 1994), and supports the view that interhemispheric motor communication occurs predominantly at the level of movement planning. Within the two subdivisions of premotor cortex, PMd exhibited greater connectivity with non-homologous regions in the opposite hemisphere (in terms of quantity of streamlines and fibre density) than PMv. To the extent that structural connectivity hints at functional correlates, the present findings suggest that PMd assumes a more prominent role than PMv in mediating bilateral interactions within the cortical motor network. They are also in accordance with the supposition that the dorsal and ventral premotor areas belong to separate interhemispheric circuits (Boussaoud et al. 2005).

In contrast with anatomical tracer studies, tractography is incapable of establishing whether structural connections provide afferent or efferent input, to or from a target region. This caveat notwithstanding, the present findings provide the most comprehensive representation of in vivo transcallosal motor network connectivity thus far derived for humans. As knowledge concerning the functional subdivisions of human motor cortical regions continues to advance, it is likely that further differentiation of transcallosal white matter connections will ultimately be revealed. While the present findings provide a unique in vivo characterization of the cortical motor network, they also demonstrate the general utility of CSD based tractography with respect to regions that comprise complex fibre architectures. The utility of these methods also extends readily to the clinical domain, particularly in relation to the identification of incipient neurodegeneration. The data presented herein provide insights in relation to the characteristics of these tracts in vivo, and delineate unique profiles of anatomical connectivity for several key brain regions in the motor network. It is anticipated that this detailed knowledge of transcallosal circuitry will serve to inform the development of future hypotheses and computational models relating structural and functional brain connectivity.

## Electronic supplementary material

Below is the link to the electronic supplementary material.

Supplementary Figure 1 Balloon plot illustrating FA values obtained for interhemispheric fibre bundles. The “greater than” symbol indicates that the connections represented by the larger (*coloured*) circle exhibit median FA values that are greater than those represented by all successive smaller (coloured) circles. The corresponding inferential analyses are reported in Supplementary Table 3 for pairwise comparisons of tracts connecting homologous regions and in Supplementary Table 6 for non-homologous comparisons. (EPS 53 kb)

Supplementary Movie 1 Tracts reconstructed using DTI based tractography, for PMd-PMd homologous connections for one representative subject. Tracts are displayed on the individual’s T1 structural image. (MP4 3929 kb)

Supplementary Movie 2 Tracts reconstructed using CSD based tractography, for PMd-PMd homologous connections for one representative subject. Tracts are displayed on the individual’s T1 structural image (MP4 3945 kb)

Supplementary material 4 (PDF 387 kb)
