# Supplementary material for: Transcallosal connectivity of the human cortical motor network
Source: Brain Struct Funct. 2016 Jul 28;222(3):1243–52. doi: 10.1007/s00429-016-1274-1 (PMC5368198; doi:10.1007/s00429-016-1274-1)
Supplement: Supplementary file 4 — Supplementary material 4 (PDF 387 kb) [file 429_2016_1274_MOESM4_ESM.pdf]

Title: Transcallosal connectivity of the human cortical motor network

Authors: Kathy L. Ruddy[1,2,3]*, Alexander Leemans[4], Richard G. Carson[1,2]

Supplementary Materials

Supplementary Figure and Movie captions:

Supplementary Figure 1- Balloon plot illustrating FA values obtained for interhemispheric fibre bundles. The "greater than" symbol indicates that the connections represented by the larger (coloured) circle exhibit median FA values that are greater than those represented by all successive smaller (coloured) circles. The corresponding inferential analyses are reported in Supplementary Table 3 for pairwise comparisons of tracts connecting homologous regions and in Supplementary Table 6 for non-homologous comparisons.

Supplementary Movie 1: Tracts reconstructed using DTI based tractography, for PMd-PMd homologous connections for one representative subject. Tracts are displayed on the individual's T1 structural image.

Supplementary Movie 2: Tracts reconstructed using CSD based tractography, for PMd-PMd homologous connections for one representative subject. Tracts are displayed on the individual's T1 structural image.

Supplementary Table 1. Median values for DWI derived indices of structural connectivity. Data are presented separately for counts of normalized reconstructed streamlines, fractional anisotropy (FA) and apparent fibre density (AFD) in fibre tracts connecting each node of the cortical motor network in the left (L) hemisphere to the corresponding nodes in the right (R) hemisphere. 95% confidence intervals generated using bootstrapping are displayed below each value. The symbol X denotes instances in which no tracts were detected (i.e. in any participant). In some other cases the estimates of AFD and FA are based upon fibre bundles wherein only a small number of streamlines were reconstructed, or upon streamlines that were reconstructed for a subset of participants, thus no confidence intervals could be computed.

Normalised Number of Streamlines

	R SMAproper	R pre-SMA	R CMA	R M1a	R M1p	R PMd	R PMv	R S1
**L SMA proper**	18.85	7.65	4.33	0.12	X	5.98	2.23	0.10
	(16.06 - 20.05)	*(5.30 - 9.92)*	*(2.63 - 5.44)*	*(0.10 - 0.21)*	*NA*	*(4.72 - 6.49)*	*(1.23 - 3.03)*	*(0.04 - 0.13)*
**L pre-SMA**	3.56	16.36	3.85	0.05	X	2.57	1.20	0.04
	(2.60 - 4.32)	*(13.53 - 17.82)*	*(2.02 - 4.47)*	*NA*	*NA*	*(1.94 - 3.21)*	*(0.77 - 1.81)*	*(0.043 - 0.044)*
**L CMA**	3.71	3.17	14.70	1.89	0.36	1.84	1.17	0.48
	(2.42 - 4.63)	*(2.02 - 3.78)*	*(12.5 - 15.83)*	*(1.34 - 2.89)*	*(0.07 - 0.51)*	*(1.29 - 2.34)*	*(0.65 - 1.60)*	*(0.30 - 0.83)*
**L M1a**	0.19	0.07	2.85	14.69	0.54	3.70	0.21	4.39
	(0.10 - 0.45)	*(0.05 - 0.17)*	*(1.84 - 3.60)*	*(13.65 - 15.56)*	*(0.41 - 1.13)*	*(2.79 - 4.04)*	*(0.09 - 0.27)*	*(3.53 - 6.02)*
**L M1p**	0.13	X	X	0.33	0.96	0.38	1.19	0.18
	NA	*NA*	*NA*	*(0.12 - 0.94)*	*(0.59 - 2.05)*	*(0.22 - 0.59)*	*(0.53 - 2.01)*	*(0.09 - 0.60)*
**L PMd**	5.49	4.90	2.32	3.27	0.18	18.19	0.88	0.32
	(4.76 - 5.81)	*(3.85 - 5.40)*	*(1.81 - 2.54)*	*(2.38 - 3.96)*	*(0.04 - 0.52)*	*(15.94 - 20.68)*	*(0.56 - 1.08)*	*(0.18 - 0.58)*
**L PMv**	2.30	1.19	1.04	0.27	0.85	1.35	2.19	0.24
	(1.55 - 3.12)	*(0.70 - 1.65)*	*(0.66 - 1.49)*	*(0.06 - 0.74)*	*(0.08 - 0.98)*	*(0.75 - 1.59)*	*(1.00 - 3.31)*	*(0.07 - 0.55)*
**L S1**	X	0.34	X	1.17	0.49	0.48	0.17	1.58
	NA	*NA*	*NA*	*(0.57 - 1.77)*	*(0.26 - 1.40)*	*(0.27 - 0.62)*	*(0.07 - 0.34)*	*(0.75 - 2.27)*

Fractional anisotropy

	R SMAproper	R pre-SMA	R CMA	R M1a	R M1p	R PMd	R PMv	R S1
**L SMA**	0.48	0.48	0.40	0.46	X	0.51	0.42	0.30

**proper**	*(0.47 - 0.49)*	*(0.47 - 0.50)*	*(0.39 - 0.41)*	*(0.30 - 0.49)*	*NA*	*(0.49 - 0.52)*	*(0.40 - 0.44)*	*(0.25 - 0.32)*
**L pre-SMA**	0.46	0.47	0.40	0.26	X	0.51	0.40	0.36
	(0.44 - 0.48)	*(0.46 - 0.48)*	*(0.37 - 0.40)*	*NA*	*NA*	*(0.50 - 0.52)*	*(0.38 - 0.43)*	*(0.25 - 0.36)*
**L CMA**	0.39	0.39	0.37	0.39	0.41	0.42	0.35	0.39
	(0.38 - 0.41)	*(0.36 - 0.40)*	*(0.35 - 0.37)*	*(0.38 - 0.40)*	*(0.35 - 0.44)*	*(0.40 - 0.43)*	*(0.33 - 0.38)*	*(0.39 - 0.40)*
**L M1a**	0.46	0.42	0.39	0.47	0.44	0.50	0.33	0.46
	(0.38 - 0.49)	*(0.34 - 0.43)*	*(0.38 - 0.40)*	*(0.46 - 0.48)*	*(0.42 - 0.46)*	*(0.48 - 0.50)*	*(0.31 - 0.37)*	*(0.44 - 0.46)*
**L M1p**	0.41	X	X	0.36	0.33	0.34	0.30	0.35
	NA	*NA*	*NA*	*(0.34 - 0.42)*	*(0.30 - 0.35)*	*(0.32 - 0.36)*	*(0.27 - 0.31)*	*(0.30 - 0.39)*
**L PMd**	0.51	0.51	0.42	0.49	0.37	0.49	0.40	0.43
	(0.50 - 0.51)	*(0.50 - 0.52)*	*(0.41 - 0.42)*	*(0.48 - 0.50)*	*(0.35 - 0.40)*	*(0.49 - 0.51)*	*(0.39 - 0.42)*	*(0.40 - 0.48)*
**L PMv**	0.43	0.41	0.38	0.34	0.31	0.39	0.35	0.31
	(0.41 - 0.44)	*(0.39 - 0.44)*	*(0.35 - 0.39)*	*(0.29 - 0.35)*	*(0.22 - 0.33)*	*(0.37 - 0.41)*	*(0.33 - 0.38)*	*(0.25 - 0.32)*
**L S1**	X	0.50	X	0.45	0.33	0.38	0.30	0.42
	NA	*NA*	*NA*	*(0.42 - 0.47)*	*(0.29 - 0.39)*	*(0.35 - 0.41)*	*(0.27 - 0.31)*	*(0.39 - 0.45)*

Apparent Fibre Density

	**R SMAproper**	**R pre-SMA**	**R CMA**	**R M1a**	**R M1p**	**R PMd**	**R PMv**	**R S1**
**L SMA proper**	0.82	0.81	0.68	0.82	X	0.86	0.71	0.41
	(0.79 - 0.83)	*(0.78 - 0.83)*	*(0.66 - 0.70)*	*(0.58 - 0.88)*	*NA*	*(0.84 - 0.87)*	*(0.66 - 0.75)*	*(0.35 - 0.42)*
**L pre-SMA**	0.77	0.77	0.65	0.47	X	0.84	0.65	0.56
	(0.74 - 0.79)	*(0.76 - 0.79)*	*(0.60 - 0.67)*	*NA*	*NA*	*(0.81 - 0.87)*	*(0.59 - 0.69)*	*(0.42 - 0.56)*
**L CMA**	0.67	0.62	0.58	0.65	0.70	0.70	0.56	0.64
	(0.61 - 0.69)	*(0.59 - 0.64)*	*(0.55 - 0.60)*	*(0.62 - 0.72)*	*(0.58 - 0.76)*	*(0.67 - 0.71)*	*(0.54 - 0.61)*	*(0.62 - 0.68)*
**L M1a**	0.77	0.67	0.68	0.85	0.78	0.90	0.56	0.81
	(0.81 - 0.86)	*(0.39 - 0.72)*	*(0.63 - 0.69)*	*(0.81 - 0.86)*	*(0.72 - 0.84)*	*(0.87 - 0.93)*	*(0.45 - 0.61)*	*(0.77 - 0.82)*
**L M1p**	0.72	X	X	0.57	0.52	0.56	0.50	0.59
	NA	*NA*	*NA*	*(0.50 - 0.74)*	*(0.43 - 0.55)*	*(0.54 - 0.58)*	*(0.44 - 0.52)*	*(0.45 - 0.63)*
**L PMd**	0.86	0.85	0.71	0.90	0.61	0.86	0.67	0.76
	(0.84 - 0.88)	*(0.82 - 0.86)*	*(0.69 - 0.74)*	*(0.88 - 0.92)*	*(0.56 - 0.68)*	*(0.85 - 0.88)*	*(0.65 - 0.70)*	*(0.70 - 0.84)*
**L PMv**	0.72	0.70	0.60	0.57	0.52	0.65	0.58	0.53
	(0.68 - 0.74)	*(0.63 - 0.72)*	*(0.56 - 0.63)*	*(0.48 - 0.58)*	*(0.33 - 0.54)*	*(0.62 - 0.69)*	*(0.56 - 0.60)*	*(0.37 - 0.58)*
**L S1**	X	0.77	X	0.79	0.52	0.63	0.49	0.70
	NA	*NA*	*NA*	*(0.72 - 0.82)*	*(0.41 - 0.56)*	*(0.58 - 0.68)*	*(0.43 - 0.51)*	*(0.61 - 0.75)*

Supplementary Table 2. Outcomes of planned contrasts for apparent fibre density (AFD) values derived for tracts projecting between homologous regions of interest. Pairwise comparisons using least squared means were conducted in the context of a mixed effects model based upon rank transformed AFD values. Following FDR correction for multiple comparisons, instances of p values smaller than the adjusted alpha level are highlighted in bold font. Each contrast is labeled with reference to the pair of homologous tracts from which it is composed. For example, 'CMA – M1a' represents the comparison between AFD values derived for CMA-CMA homologous tracts and AFD values derived for M1a-M1a homologous tracts.

**Contrast**	**df**	**t**	**p**
CMA - M1a	252.057	-13.575	**<0.001**
CMA - M1p	268.941	1.518	0.130
CMA - PMd	252.057	-15.213	**<0.001**
CMA - PMv	253.619	-1.867	0.068
CMA - preSMA	252.057	-8.770	**<0.001**
CMA - S1	254.216	-5.114	**<0.001**
CMA - SMAproper	252.057	-11.112	**<0.001**
M1a - M1p	268.941	9.643	**<0.001**
M1a - PMd	252.057	-1.638	0.106
M1a - PMv	253.619	11.345	**<0.001**
M1a - preSMA	252.057	4.805	**<0.001**
M1a - S1	254.216	7.998	**<0.001**

M1a - SMAproper	252.057	2.462	**0.017**
M1p - PMd	268.941	-10.623	**<0.001**
M1p - PMv	271.140	-2.630	**0.011**
M1p - preSMA	268.941	-6.767	**<0.001**
M1p - S1	267.925	-4.637	**<0.001**
M1p - SMAproper	268.941	-8.169	**<0.001**
PMd - PMv	253.619	12.939	**<0.001**
PMd - preSMA	252.057	6.443	**<0.001**
PMd - S1	254.216	9.581	**<0.001**
PMd - SMAproper	252.057	4.101	**<0.001**
PMv - preSMA	253.619	-6.669	**<0.001**
PMv - S1	256.019	-3.178	**0.002**
PMv - SMAproper	253.619	-8.948	**<0.001**
preSMA - S1	254.216	3.357	**0.001**
preSMA - SMAproper	252.057	-2.342	**0.022**
S1 - SMAproper	254.216	-5.620	**<0.001**

Supplementary Table 3. Outcomes of planned contrasts for fractional anisotropy (FA) values derived for tracts projecting between homologous regions of interest. Pairwise comparisons using least squared means were conducted in the context of a mixed effects model based upon rank transformed FA values. Following FDR correction for multiple comparisons, instances of p values smaller than the adjusted alpha level are highlighted in bold font. Each contrast is labeled

with reference to the pair of homologous tracts from which it is composed. For example, ‘CMA – M1a’ represents the comparison between FA values derived for CMA-CMA homologous tracts and FA values derived for M1a-M1a homologous tracts.

**Contrast**	**df**	**t**	**p**
CMA - M1a	252.034	-10.852	**<0.001**
CMA - M1p	265.436	1.556	0.130
CMA - PMd	252.034	-15.153	**<0.001**
CMA - PMv	253.257	-1.840	0.075
CMA - preSMA	252.034	-11.228	**<0.001**
CMA - S1	253.754	-5.267	**<0.001**
CMA - SMAproper	252.034	-12.751	**<0.001**
M1a - M1p	265.436	8.024	**<0.001**
M1a - PMd	252.034	-4.301	**<0.001**
M1a - PMv	253.257	8.718	**<0.001**
M1a - preSMA	252.034	-0.376	0.707
M1a - S1	253.754	5.210	**<0.001**
M1a - SMAproper	252.034	-1.899	0.069
M1p - PMd	265.436	-10.588	**<0.001**
M1p - PMv	267.295	-2.646	**0.011**
M1p - preSMA	265.436	-8.248	**<0.001**
M1p - S1	264.583	-4.759	**<0.001**
M1p - SMAproper	265.436	-9.156	**<0.001**

PMd - PMv	253.257	12.902	**<0.001**
PMd - preSMA	252.034	3.925	**<0.001**
PMd - S1	253.754	9.362	**<0.001**
PMd - SMAproper	252.034	2.402	**0.021**
PMv - preSMA	253.257	-9.084	**<0.001**
PMv - S1	255.185	-3.352	**0.001**
PMv - SMAproper	253.257	-10.565	**<0.001**
preSMA - S1	253.754	5.573	**<0.001**
preSMA - SMAproper	252.034	-1.523	0.134
S1 - SMAproper	253.754	-7.043	**<0.001**

Supplementary Table 4. Mean diffusivity and radial diffusivity values in mm^2/s. Data are presented separately for fibre tracts connecting each node of the cortical motor network in the left (L) hemisphere to the corresponding nodes in the right (R) hemisphere. The symbol X denotes instances in which no tracts were detected (i.e. in any participant). In some other cases the estimates are based upon fibre bundles wherein only a small number of streamlines were reconstructed, or upon streamlines that were reconstructed for a subset of participants

(Supplementary Table 1). Due to the nature of the extremely small numbers derived for both of these indices, all values have been multiplied by a factor of 10^8.

**Mean Diffusivity**								
	R SMAproper	R pre-SMA	R CMA	R M1a	R M1p	R PMd	R PMv	R S1
L SMAproper	69180.0	69707.0	69051.0	71208.0	X	69186.0	69994.0	66934.0
L pre-SMA	70015.5	70362.0	70668.0	73191.0	X	70538.0	71211.5	72324.5
L CMA	69000.0	70097.0	71127.0	69706.0	70933.0	68721.0	70301.0	69888.5
L M1a	69412.0	71013.0	69697.0	69682.0	71045.5	69154.0	70408.0	70143.0
L M1p	72616.0	X	X	71392.0	70320.0	69917.0	70657.0	72429.0
L PMd	69185.0	70036.0	69136.0	69144.0	70037.5	69476.0	69486.0	70138.0
L PMv	69388.0	70629.0	70024.0	70394.0	72296.0	69531.0	69285.0	71189.0
L S1	X	71637.0	X	71583.0	71909.0	70731.0	70894.0	72128.0
**Radial Diffusivity**								
	R SMAproper	R pre-SMA	R CMA	R M1a	R M1p	R PMd	R PMv	R S1
L SMAproper	48335.0	48414.5	53563.5	52392.0	X	47215.0	52306.0	56953.0
L pre-SMA	50144.0	49520.0	54481.0	62695.0	X	47911.0	54059.0	55862.5
L CMA	53721.0	54990.0	57117.0	54274.5	53107.0	52884.0	56625.0	54665.5
L M1a	50078.5	53202.0	54383.0	49851.0	51951.0	49676.0	55952.0	50853.0
L M1p	58760.0	X	X	55502.5	56932.0	56356.0	58778.0	57308.0
L PMd	46850.0	47396.0	52468.0	49088.0	55838.0	48756.0	53626.0	52312.0
L PMv	52463.0	53474.5	54906.0	57266.0	59669.0	53643.0	55187.0	58863.0
L S1	X	51290.0	X	53416.5	57897.0	56809.0	59220.0	54070.0

Supplementary Table 5. Outcomes of planned contrasts for apparent fibre density (AFD) values derived for tracts projecting between non-homologous regions of interest. Pairwise comparisons using least squared means were conducted in the context of a mixed effects model based upon rank transformed AFD values. Following FDR correction for multiple comparisons, instances of p values smaller than the adjusted alpha level are highlighted in bold font. Under each "Target region" subsection of the table, each contrast is labeled with reference to the source ROIs (i.e. those projecting to the target ROI) for the two tracts being compared. For example, in the Target region: CMA subsection, 'CMA – M1a' represents the comparison between the AFD values derived for CMA-CMA tracts and the AFD values derived for CMA-M1a tracts.

Target region: CMA			
Contrast	df	t.ratio	p.value
CMA - M1a	246.004	-6.670	**<0.001**
CMA - M1p	251.889	-2.807	**0.010**
CMA - PMd	246.004	-10.545	**<0.001**
CMA - PMv	246.102	-0.938	0.425
CMA - preSMA	246.004	-4.267	**<0.001**
CMA - S1	247.190	-4.077	**<0.001**
CMA - SMAproper	246.004	-6.159	**<0.001**
M1a - M1p	251.889	0.091	0.927
M1a - PMd	246.004	-3.875	**<0.001**
M1a - PMv	246.102	5.688	**<0.001**
M1a - preSMA	246.004	2.403	**0.029**
M1a - S1	247.190	2.138	0.052
M1a - SMAproper	246.004	0.511	0.683
M1p - PMd	251.889	-1.775	0.108
M1p - PMv	252.186	2.388	**0.029**
M1p - preSMA	251.889	0.953	0.425
M1p - S1	251.506	0.897	0.433

M1p - SMAproper	251.889	0.131	0.927
PMd - PMv	246.102	9.536	**<0.001**
PMd - preSMA	246.004	6.277	**<0.001**
PMd - S1	247.190	5.749	**<0.001**
PMd - SMAproper	246.004	4.385	**<0.001**
PMv - preSMA	246.102	-3.301	**0.002**
PMv - S1	247.329	-3.177	**0.003**
PMv - SMAproper	246.102	-5.181	**<0.001**
preSMA - S1	247.190	-0.100	0.927
preSMA - SMAproper	246.004	-1.892	0.088
S1 - SMAproper	247.190	-1.663	0.130
Target region: M1a			
Contrast	df	t.ratio	p.value
CMA - M1a	219.122	-9.757	**<0.001**
CMA - M1p	223.229	-3.157	**0.003**
CMA - PMd	219.122	-11.231	**<0.001**
CMA - PMv	223.804	1.422	0.175
CMA - preSMA	231.451	0.425	0.696
CMA - S1	219.122	-6.738	**<0.001**
CMA - SMAproper	223.448	-3.290	**0.002**
M1a - M1p	223.229	5.592	**<0.001**
M1a - PMd	219.122	-1.474	0.166
M1a - PMv	223.804	10.072	**<0.001**
M1a - preSMA	231.451	5.686	**<0.001**
M1a - S1	219.122	3.020	**0.004**
M1a - SMAproper	223.448	5.456	**<0.001**
M1p - PMd	223.229	-6.914	**<0.001**
M1p - PMv	226.293	4.161	**<0.001**
M1p - preSMA	232.686	2.240	**0.032**
M1p - S1	223.229	-2.885	**0.006**
M1p - SMAproper	226.624	-0.123	0.903
PMd - PMv	223.804	11.378	**<0.001**
PMd - preSMA	231.451	6.480	**<0.001**

PMd - S1	219.122	4.493	**<0.001**
PMd - SMAproper	223.448	6.777	**<0.001**
PMv - preSMA	230.599	-0.425	0.696
PMv - S1	223.804	-7.395	**<0.001**
PMv - SMAproper	223.893	-4.310	**<0.001**
preSMA - S1	231.451	-4.058	**<0.001**
preSMA - SMAproper	230.790	-2.332	**0.026**
S1 - SMAproper	223.448	2.750	**0.009**
Target region: M1p			
Contrast	df	t.ratio	p.value
CMA - M1a	82.619	0.238	0.893
CMA - M1p	81.716	3.413	**0.004**
CMA - PMd	83.245	3.100	**0.008**
CMA - PMv	82.958	4.145	**<0.001**
CMA - S1	82.448	2.602	**0.026**
CMA - SMAproper	85.705	0.189	0.893
M1a - M1p	83.516	4.773	**<0.001**
M1a - PMd	83.812	5.750	**<0.001**
M1a - PMv	82.392	6.610	**<0.001**
M1a - S1	81.089	4.444	**<0.001**
M1a - SMAproper	85.592	0.095	0.925
M1p - PMd	83.635	-1.010	0.420
M1p - PMv	82.919	0.766	0.520
M1p - S1	83.049	-1.521	0.224
M1p - SMAproper	85.969	-1.494	0.224
PMd - PMv	84.146	2.198	**0.064**
PMd - S1	81.787	-0.825	0.509
PMd - SMAproper	85.788	-1.211	0.344
PMv - S1	82.819	-2.708	**0.022**
PMv - SMAproper	84.875	-1.831	0.135
S1 - SMAproper	86.049	-1.001	0.420
Target region: PMd			

Contrast	df	t.ratio	p.value
CMA - M1a	287.006	-11.846	**<0.001**
CMA - M1p	288.648	6.134	**<0.001**
CMA - PMd	287.006	-11.154	**<0.001**
CMA - PMv	287.006	1.423	0.190
CMA - preSMA	287.006	-8.833	**<0.001**
CMA - S1	287.006	0.390	0.697
CMA - SMAproper	287.006	-10.700	**<0.001**
M1a - M1p	288.648	17.389	**<0.001**
M1a - PMd	287.006	0.693	0.527
M1a - PMv	287.006	13.269	**<0.001**
M1a - preSMA	287.006	3.013	**0.004**
M1a - S1	287.006	12.236	**<0.001**
M1a - SMAproper	287.006	1.147	0.295
M1p - PMd	288.648	-16.731	**<0.001**
M1p - PMv	288.648	-4.782	**<0.001**
M1p - preSMA	288.648	-14.526	**<0.001**
M1p - S1	288.648	-5.763	**<0.001**
M1p - SMAproper	288.648	-16.300	**<0.001**
PMd - PMv	287.006	12.576	**<0.001**
PMd - preSMA	287.006	2.320	**0.028**
PMd - S1	287.006	11.544	**<0.001**
PMd - SMAproper	287.006	0.454	0.674
PMv - preSMA	287.006	-10.256	**<0.001**
PMv - S1	287.006	-1.033	0.339
PMv - SMAproper	287.006	-12.123	**<0.001**
preSMA - S1	287.006	9.223	**<0.001**
preSMA - SMAproper	287.006	-1.866	0.080
S1 - SMAproper	287.006	-11.090	**<0.001**
Target region: PMv			
Contrast	df	t.ratio	p.value
CMA - M1a	219.885	-1.724	0.105
CMA - M1p	225.089	2.210	**0.041**

CMA - PMd	215.783	-4.916	**<0.001**
CMA - PMv	215.947	-1.791	0.096
CMA - preSMA	216.685	-3.621	**0.001**
CMA - S1	224.945	1.956	0.072
CMA - SMAproper	216.575	-6.383	**<0.001**
M1a - M1p	222.649	3.401	**0.002**
M1a - PMd	220.372	-2.657	**0.013**
M1a - PMv	221.088	0.089	0.929
M1a - preSMA	221.754	-1.539	0.140
M1a - S1	223.046	3.176	**0.003**
M1a - SMAproper	220.534	-4.004	**<0.001**
M1p - PMd	225.399	-5.676	**<0.001**
M1p - PMv	226.785	-3.448	**0.002**
M1p - preSMA	226.490	-4.757	**<0.001**
M1p - S1	219.823	-0.256	0.828
M1p - SMAproper	225.055	-6.735	**<0.001**
PMd - PMv	216.502	3.014	**0.005**
PMd - preSMA	216.038	1.204	0.248
PMd - S1	225.287	5.508	**<0.001**
PMd - SMAproper	215.950	-1.544	0.140
PMv - preSMA	216.838	-1.786	0.096
PMv - S1	226.578	3.228	**0.003**
PMv - SMAproper	217.347	-4.476	**<0.001**
preSMA - S1	227.123	4.558	**<0.001**
preSMA - SMAproper	216.166	-2.705	**0.012**
S1 - SMAproper	225.723	-6.579	**<0.001**
Target region: PreSMA			
Contrast	df	t.ratio	p.value
CMA - M1a	179.804	0.263	0.877
CMA - PMd	174.011	-13.950	<0.001
CMA - PMv	174.521	-1.830	0.097
CMA - preSMA	174.011	-10.202	**<0.001**
CMA - S1	180.693	0.160	0.917

CMA - SMAproper	174.011	-9.785	**<0.001**
M1a - PMd	179.804	-7.657	**<0.001**
M1a - PMv	179.614	-1.249	0.280
M1a - preSMA	179.804	-5.671	**<0.001**
M1a - S1	180.332	-0.007	0.995
M1a - SMAproper	179.804	-5.449	**<0.001**
PMd - PMv	174.521	11.814	**<0.001**
PMd - preSMA	174.011	3.748	**<0.001**
PMd - S1	180.693	4.873	**<0.001**
PMd - SMAproper	174.011	4.165	**<0.001**
PMv - preSMA	174.521	-8.149	**<0.001**
PMv - S1	180.613	0.791	0.531
PMv - SMAproper	174.521	-7.740	**<0.001**
preSMA - S1	180.693	3.607	**0.001**
preSMA - SMAproper	174.011	0.418	0.789
S1 - SMAproper	180.693	-3.466	**0.001**
Target region: S1			
Contrast	df	t.ratio	p.value
CMA - M1a	159.051	-6.798	**<0.001**
CMA - M1p	161.566	0.725	0.597
CMA - PMd	159.051	-2.255	**0.045**
CMA - PMv	162.626	3.075	**0.007**
CMA - preSMA	165.010	-0.172	0.879
CMA - S1	159.175	-1.921	0.088
CMA - SMAproper	167.177	2.054	0.068
M1a - M1p	161.562	6.796	**<0.001**
M1a - PMd	156.250	4.894	**<0.001**
M1a - PMv	163.202	8.364	**<0.001**
M1a - preSMA	165.400	2.314	**0.042**
M1a - S1	158.186	4.991	**<0.001**
M1a - SMAproper	167.050	5.219	**<0.001**
M1p - PMd	161.562	-2.762	**0.015**
M1p - PMv	161.750	2.304	**0.042**

M1p - preSMA	165.380	-0.474	0.712
M1p - S1	160.062	-2.476	**0.031**
M1p - SMAproper	167.846	1.614	0.152
PMd - PMv	163.202	4.897	**<0.001**
PMd - preSMA	165.400	0.652	0.627
PMd - S1	158.186	0.285	0.835
PMd - SMAproper	167.050	3.124	**0.007**
PMv - preSMA	164.756	-1.617	0.152
PMv - S1	162.608	-4.615	**<0.001**
PMv - SMAproper	165.693	0.152	0.879
preSMA - S1	165.165	-0.550	0.681
preSMA - SMAproper	165.380	1.499	0.181
S1 - SMAproper	167.188	2.972	**0.009**
Target Region SMA proper			
Contrast	df	t.ratio	p.value
CMA - M1a	202.931	-4.306	**<0.001**
CMA - M1p	216.455	-1.265	0.264
CMA - PMd	199.047	-12.077	**<0.001**
CMA - PMv	199.622	-3.433	**0.001**
CMA - preSMA	199.047	-6.975	**<0.001**
CMA - S1	212.230	3.526	**0.001**
CMA - SMAproper	199.047	-9.474	**<0.001**
M1a - M1p	217.077	-0.300	0.793
M1a - PMd	202.931	-6.509	**<0.001**
M1a - PMv	202.940	1.177	0.292
M1a - preSMA	202.931	-1.940	0.072
M1a - S1	211.860	5.501	**<0.001**
M1a - SMAproper	202.931	-4.178	**<0.001**
M1p - PMd	216.455	-1.153	0.292
M1p - PMv	216.416	0.568	0.615
M1p - preSMA	216.455	-0.132	0.895
M1p - S1	216.807	2.662	**0.013**
M1p - SMAproper	216.455	-0.632	0.591

PMd - PMv	199.622	8.479	**<0.001**
PMd - preSMA	199.047	5.103	**<0.001**
PMd - S1	212.230	8.775	**<0.001**
PMd - SMAproper	199.047	2.603	**0.015**
PMv - preSMA	199.622	-3.446	**0.001**
PMv - S1	212.754	5.015	**<0.001**
PMv - SMAproper	199.622	-5.912	**<0.001**
preSMA - S1	212.230	6.557	**<0.001**
preSMA - SMAproper	199.047	-2.500	**0.019**
S1 - SMAproper	212.230	-7.644	**<0.001**

Supplementary Table 6. Outcomes of planned contrasts for fractional anisotropy (FA) values derived for tracts projecting between non-homologous regions of interest. Pairwise comparisons using least squared means were conducted in the context of a mixed effects model based upon rank transformed FA values. Following FDR correction for multiple comparisons, instances of p values smaller than the adjusted alpha level are highlighted in bold font. Under each "Target region" subsection of the table, each contrast is labeled with reference to the source ROIs (i.e. those projecting to the target ROI) for the two tracts being compared. For example, in the Target region: CMA subsection, 'CMA – M1a' represents the comparison between the FA values derived for CMA-CMA tracts and the FA values derived for CMA-M1a tracts.

**Target region: SMA proper**			
Contrast	df	t	p
CMA - M1a	202.948	-3.714	**<0.001**
CMA - M1p	216.522	-0.577	0.632
CMA - PMd	199.047	-13.059	**<0.001**
CMA - PMv	199.625	-4.026	**<0.001**
CMA - preSMA	199.047	-8.401	**<0.001**
CMA - S1	212.286	2.166	**0.046**

CMA - SMAproper	199.047	-10.280	**<0.001**
M1a - M1p	217.146	0.251	0.841
M1a - PMd	202.948	-7.980	**<0.001**
M1a - PMv	202.956	0.059	0.953
M1a - preSMA	202.948	-3.808	**<0.001**
M1a - S1	211.914	3.888	**<0.001**
M1a - SMAproper	202.948	-5.491	**<0.001**
M1p - PMd	216.522	-2.037	0.060
M1p - PMv	216.483	-0.240	0.841
M1p - preSMA	216.522	-1.105	0.316
M1p - S1	216.875	1.452	0.180
M1p - SMAproper	216.522	-1.481	0.178
PMd - PMv	199.625	8.855	**<0.001**
PMd - preSMA	199.047	4.658	**<0.001**
PMd - S1	212.286	7.842	**<0.001**
PMd - SMAproper	199.047	2.779	**0.009**
PMv - preSMA	199.625	-4.260	**<0.001**
PMv - S1	212.811	3.922	**<0.001**
PMv - SMAproper	199.625	-6.114	**<0.001**
preSMA - S1	212.286	5.817	**<0.001**
preSMA - SMAproper	199.047	-1.879	0.082
S1 - SMAproper	212.286	-6.634	**<0.001**
**Target region: Pre-SMA**			
Contrast	df	t	p
CMA - M1a	182.712	-0.802	0.468
CMA - PMd	174.025	-15.085	**<0.001**
CMA - PMv	174.764	-2.221	**0.041**
CMA - preSMA	174.025	-10.905	**<0.001**
CMA - S1	184.082	-1.436	0.200
CMA - SMAproper	174.025	-10.084	**<0.001**
M1a - PMd	182.712	-7.229	**<0.001**
M1a - PMv	182.437	-0.405	0.686
M1a - preSMA	182.712	-5.004	**<0.001**

M1a - S1	183.552	-0.818	0.468
M1a - SMAproper	182.712	-4.567	**<0.001**
PMd - PMv	174.764	12.539	**<0.001**
PMd - preSMA	174.025	4.180	**<0.001**
PMd - S1	184.082	3.686	**<0.001**
PMd - SMAproper	174.025	5.001	**<0.001**
PMv - preSMA	174.764	-8.449	**<0.001**
PMv - S1	183.967	-0.664	0.533
PMv - SMAproper	174.764	-7.646	**<0.001**
preSMA - S1	184.082	2.267	**0.040**
preSMA - SMAproper	174.025	0.821	0.468
S1 - SMAproper	184.082	-1.988	0.068
**Target regon: CMA**			
Contrast	df	t	p
CMA - M1a	246.005	-4.736	**<0.001**
CMA - M1p	252.293	-3.118	**0.004**
CMA - PMd	246.005	-9.765	**<0.001**
CMA - PMv	246.110	-1.291	0.326
CMA - preSMA	246.005	-4.682	**<0.001**
CMA - S1	247.270	-4.541	**<0.001**
CMA - SMAproper	246.005	-5.651	**<0.001**
M1a - M1p	252.293	-1.059	0.407
M1a - PMd	246.005	-5.029	**<0.001**
M1a - PMv	246.110	3.413	**0.002**
M1a - preSMA	246.005	0.054	0.957
M1a - S1	247.270	-0.128	0.932
M1a - SMAproper	246.005	-0.915	0.440
M1p - PMd	252.293	-1.127	0.406
M1p - PMv	252.609	2.543	**0.020**
M1p - preSMA	252.293	1.083	0.407
M1p - S1	251.886	0.989	0.424
M1p - SMAproper	252.293	0.661	0.570
PMd - PMv	246.110	8.409	**<0.001**

PMd - preSMA	246.005	5.083	**<0.001**
PMd - S1	247.270	4.559	**<0.001**
PMd - SMAproper	246.005	4.114	**<0.001**
PMv - preSMA	246.110	-3.359	**0.002**
PMv - S1	247.419	-3.309	**0.002**
PMv - SMAproper	246.110	-4.322	**<0.001**
preSMA - S1	247.270	-0.178	0.925
preSMA - SMAproper	246.005	-0.970	0.424
S1 - SMAproper	247.270	-0.725	0.547
**Target region: M1a**			
Contrast	df	t	p
CMA - M1a	219.142	-9.139	**<0.001**
CMA - M1p	223.545	-3.153	**0.003**
CMA - PMd	219.142	-10.817	**<0.001**
CMA - PMv	224.155	1.923	0.068
CMA - preSMA	232.391	-0.927	0.368
CMA - S1	219.142	-7.149	**<0.001**
CMA - SMAproper	223.772	-3.778	**<0.001**
M1a - M1p	223.545	5.045	**<0.001**
M1a - PMd	219.142	-1.678	0.111
M1a - PMv	224.155	10.028	**<0.001**
M1a - preSMA	232.391	4.005	**<0.001**
M1a - S1	219.142	1.990	0.061
M1a - SMAproper	223.772	4.418	**<0.001**
M1p - PMd	223.545	-6.550	**<0.001**
M1p - PMv	226.833	4.617	**<0.001**
M1p - preSMA	233.690	0.935	0.368
M1p - S1	223.545	-3.260	**0.002**
M1p - SMAproper	227.181	-0.572	0.568
PMd - PMv	224.155	11.517	**<0.001**
PMd - preSMA	232.391	4.911	**<0.001**
PMd - S1	219.142	3.668	**<0.001**
PMd - SMAproper	223.772	5.922	**<0.001**

PMv - preSMA	231.475	-2.029	0.058
PMv - S1	224.155	-8.263	**<0.001**
PMv - SMAproper	224.272	-5.217	**<0.001**
preSMA - S1	232.391	-2.931	**0.005**
preSMA - SMAproper	231.678	-1.306	0.216
S1 - SMAproper	223.772	2.633	**0.013**
**Target region: M1p**			
Contrast	df	t	p
CMA - M1a	84.540	0.490	0.730
CMA - M1p	83.415	3.746	**<0.001**
CMA - PMd	85.266	3.955	**<0.001**
CMA - PMv	85.019	5.018	**<0.001**
CMA - S1	84.390	2.771	**0.016**
CMA - SMAproper	88.444	0.330	0.780
M1a - M1p	85.342	4.943	**<0.001**
M1a - PMd	84.873	6.965	**<0.001**
M1a - PMv	83.886	7.684	**<0.001**
M1a - S1	82.136	4.294	**<0.001**
M1a - SMAproper	88.264	0.131	0.896
M1p - PMd	85.463	-0.370	0.780
M1p - PMv	84.707	1.439	0.202
M1p - S1	84.879	-1.786	0.125
M1p - SMAproper	88.740	-1.519	0.199
PMd - PMv	85.870	2.346	**0.045**
PMd - S1	82.869	-2.112	0.066
PMd - SMAproper	88.486	-1.453	0.202
PMv - S1	84.526	-3.851	**<0.001**
PMv - SMAproper	87.393	-2.112	0.066
S1 - SMAproper	88.840	-0.932	0.437
**Target region: PMd**			
Contrast	df	t	p
CMA - M1a	287.004	-10.658	**<0.001**

CMA - M1p	288.358	8.681	**<0.001**
CMA - PMd	287.004	-12.120	**<0.001**
CMA - PMv	287.004	1.908	0.073
CMA - preSMA	287.004	-13.282	**<0.001**
CMA - S1	287.004	1.190	0.263
CMA - SMAproper	287.004	-13.501	**<0.001**
M1a - M1p	288.358	18.805	**<0.001**
M1a - PMd	287.004	-1.461	0.177
M1a - PMv	287.004	12.566	**<0.001**
M1a - preSMA	287.004	-2.623	**0.012**
M1a - S1	287.004	11.849	**<0.001**
M1a - SMAproper	287.004	-2.843	**0.007**
M1p - PMd	288.358	-20.193	**<0.001**
M1p - PMv	288.358	-6.869	**<0.001**
M1p - preSMA	288.358	-21.297	**<0.001**
M1p - S1	288.358	-7.550	**<0.001**
M1p - SMAproper	288.358	-21.505	**<0.001**
PMd - PMv	287.004	14.027	**<0.001**
PMd - preSMA	287.004	-1.162	0.265
PMd - S1	287.004	13.310	**<0.001**
PMd - SMAproper	287.004	-1.382	0.196
PMv - preSMA	287.004	-15.189	**<0.001**
PMv - S1	287.004	-0.717	0.491
PMv - SMAproper	287.004	-15.409	**<0.001**
preSMA - S1	287.004	14.472	**<0.001**
preSMA - SMAproper	287.004	-0.219	0.827
S1 - SMAproper	287.004	-14.692	**<0.001**
**Target region: PMv**			
Contrast	df	t	p
CMA - M1a	219.487	-0.208	0.867
CMA - M1p	224.241	3.095	**0.004**
CMA - PMd	215.723	-4.035	**<0.001**
CMA - PMv	215.849	-1.211	0.265

CMA - preSMA	216.554	-3.987	**<0.001**
CMA - S1	224.125	2.765	**0.009**
CMA - SMAproper	216.461	-6.444	**<0.001**
M1a - M1p	221.982	3.104	**0.004**
M1a - PMd	219.950	-3.391	**0.002**
M1a - PMv	220.595	-0.879	0.426
M1a - preSMA	221.233	-3.359	**0.002**
M1a - S1	222.353	2.781	**0.009**
M1a - SMAproper	220.101	-5.562	**<0.001**
M1p - PMd	224.537	-5.940	**<0.001**
M1p - PMv	225.813	-3.907	**<0.001**
M1p - preSMA	225.555	-5.889	**<0.001**
M1p - S1	219.392	-0.338	0.793
M1p - SMAproper	224.220	-7.656	**<0.001**
PMd - PMv	216.377	2.734	**0.009**
PMd - preSMA	215.936	-0.034	0.973
PMd - S1	224.451	5.680	**<0.001**
PMd - SMAproper	215.865	-2.481	**0.018**
PMv - preSMA	216.687	-2.718	**0.009**
PMv - S1	225.637	3.602	**<0.001**
PMv - SMAproper	217.165	-5.110	**<0.001**
preSMA - S1	226.163	5.618	**<0.001**
preSMA - SMAproper	216.060	-2.401	**0.021**
S1 - SMAproper	224.860	-7.423	**<0.001**
**Target region: S1**			
Contrast	df	t	p
CMA - M1a	159.091	-6.295	**<0.001**
CMA - M1p	161.648	1.106	0.329
CMA - PMd	159.091	-1.524	0.181
CMA - PMv	162.728	4.727	**<0.001**
CMA - preSMA	165.155	-1.460	0.195
CMA - S1	159.219	-2.439	**0.030**
CMA - SMAproper	167.341	1.657	0.146

M1a - M1p	161.640	6.749	**<0.001**
M1a - PMd	156.256	5.139	**<0.001**
M1a - PMv	163.308	9.688	**<0.001**
M1a - preSMA	165.550	0.834	0.421
M1a - S1	158.213	3.918	**<0.001**
M1a - SMAproper	167.212	4.585	**<0.001**
M1p - PMd	161.640	-2.512	**0.026**
M1p - PMv	161.840	3.549	**0.001**
M1p - preSMA	165.530	-1.899	0.092
M1p - S1	160.123	-3.333	**0.002**
M1p - SMAproper	168.020	1.031	0.345
PMd - PMv	163.308	6.047	**<0.001**
PMd - preSMA	165.550	-0.912	0.391
PMd - S1	158.213	-1.024	0.345
PMd - SMAproper	167.212	2.384	**0.032**
PMv - preSMA	164.898	-3.634	**0.001**
PMv - S1	162.710	-6.696	**<0.001**
PMv - SMAproper	165.839	-1.180	0.305
preSMA - S1	165.312	0.548	0.584
preSMA - SMAproper	165.525	2.301	**0.037**
S1 - SMAproper	167.353	2.817	**0.012**
